# Cardiovascular, respiratory and splenic responses to rebreathing and apnoea during exercise

**DOI:** 10.1113/EP093350

**Published:** 2025-12-28

**Authors:** Theodore Dotevall, Maja Persson, Bodil Sjögreen, Mats H. Linér, Angelica Lodin‐Sundström, Johan P. A. Andersson

**Affiliations:** ^1^ Department of Experimental Medical Science Lund University Lund Sweden; ^2^ Department of Biology Lund University Lund Sweden; ^3^ Department of Health Sciences Mid Sweden University Sundsvall Sweden

**Keywords:** apnoea, exercise, rebreathing

## Abstract

We investigated integrative physiological responses to eupnoeic exercise (EX), rebreathing exercise (RB), dynamic apnoea (DA) and dynamic apnoea with cold‐water face immersion (DAFI) in 20 healthy participants. Trials involved non‐steady‐state cycle exercise at 60 W for an average duration of 66 s. With increases in heart rate and stroke volume, EX and RB increased cardiac output compared with baseline (mean [SD] EX +47 [13]%, RB +43 [15]%). During DA and DAFI, the increase in cardiac output was attenuated (DA +26 [23]%, DAFI +14 [21]%). EX and RB elicited reductions in total peripheral resistance (EX −37 [7]%, RB −23 [15]%). This reduction was absent during apnoeas (DA +3 [31]%, DAFI +15 [40]%). Pulmonary oxygen uptake was the lowest during DAFI. At the end of hypoxic trials, end‐tidal partial pressures of O_2_ were RB 50.3 [11.9], DA 57.9 [14.0] and DAFI 61.4 [13.6] mmHg, indicating a preservation of the central oxygen store during DA and DAFI. At the same time, peripheral tissue oxygen saturation, measured in the working rectus femoris muscle, declined the most during DA and DAFI (RB −1.4 [3.5]%, DA −4.7 [3.3]%, DAFI −5.6 [4.4]%). Splenic volume increased during EX (+8.4 [5.8]%) but decreased during RB (−10.5 [10.2]%), DA (−6.4 [10.8]) and DAFI (−13.3 [11.1]%) when compared with EX, suggesting erythrocyte mobilization in the threat of hypoxia. The non‐steady‐state apnoea interventions of the present study evoke a progressive shift from exercise‐induced cardiovascular responses towards a diving response, including cardiac, vascular and splenic responses. These responses are amplified to some extent by cold‐water face immersion. Apnoea‐induced responses lead to central oxygen preservation and a decrease in peripheral oxygen stores.

## Abstract (highlights)

- **What is the central question of this study?**
  How does simultaneous initiation of exercise and apnoea (dynamic apnoea) affect cardiovascular, respiratory and splenic responses, and how are these responses modulated by cold‐water face immersion?
- **What is the main finding and its importance?**
  Dynamic apnoea induces a progressive shift from exercise‐induced cardiovascular responses towards a diving response. Cold‐water face immersion amplifies this shift. These changes preserve central oxygen stores at the expense of peripheral stores. The study highlights responses that prioritize oxygen conservation centrally, offering new insights into hypoxia tolerance and autonomic regulation under stress.

## 1 INTRODUCTION

At the very onset of exercise, both the heart rate (HR) and the stroke volume (SV) increase (Giuriato et al., 2024). The increases in HR and ventricular contractility with initiation of exercise are explained by a withdrawal of cardiac parasympathetic efferent activity followed by an increase in cardiac sympathetic efferent activity as exercise continues (Fagraeus & Linnarsson, 1976; Maciel et al., 1986). At the same time, with dynamic muscle contractions, the skeletal muscle pump will increase cardiac preload and, to a large extent, explain the increase in SV (Higginbotham et al., 1986). The peripheral blood flow is redistributed towards active skeletal muscle, with a reduction in visceral blood flow. How blood flow is redistributed and skeletal muscle blood flow increased is not completely understood, but the regulation involves sympathetic vasoconstriction and an integration of different vasodilator systems, such as endothelium‐dependent vasodilatation and active hyperaemia (Hellsten & Nyberg, 2015). Although exercise generally is characterized by increased activity in the sympathetic nervous system, apnoea is associated with simultaneous increases in both sympathetic and parasympathetic autonomic activity, resulting in a set of cardiovascular reflexes commonly referred to as the diving response (Gooden, 1994). Increased cardiac vagal activity during apnoea leads to bradycardia (Finley et al., 1979), while peripheral vasoconstriction follows increased sympathetic nerve activity (Leuenberger et al., 2001). The apnoeic vascular response increases systemic blood pressure and limits peripheral blood flow, for example, away from skeletal muscle, with a redistribution of blood flow towards prioritized organs, such as the brain (Palada, Obad, et al., 2007; Persson et al., 2023). In addition, the apnoea‐induced increase in sympathetic nerve activity causes a contraction of the spleen and a release of sequestered erythrocytes (Baković et al., 2005; Palada, Eterovic, et al., 2007; Schagatay et al., 2001).

Although there is knowledge about the cardiovascular responses to dynamic exercise and apnoea when performed separately, less is known about the responses to apnoea performed during exercise. With regard to steady‐state exercise combined with apnoea, there are studies on various physiological responses (e.g., Andersson et al., 2002; Bjertnaes et al., 1984; Bouten et al., 2021; Lindholm et al., 1999; Matsutake et al., 2025; Nishiyasu et al., 2012). From these, the majority of data support that apnoeic bradycardia is the dominant effect that supersedes exercise tachycardia and that apnoea during exercise is largely associated with cardiovascular responses that are similar in quality to those observed at rest. However, in many situations outside of the experimental setting in a laboratory, exercise and apnoea are initiated more or less simultaneously and not in steady‐state conditions. These are the conditions in the competitive category of ‘dynamic apnoea’ (Fitz‐Clarke, 2018) and the situations relevant for most recreational and professional freedivers (Abrahamsson & Schagatay, 2014; Shiraki et al., 2002). There is still a lack of studies focusing on integrative physiological responses to exercise and apnoea initiated simultaneously, representing non‐steady‐state conditions.

When exercise and apnoea are initiated simultaneously, there are different inputs that will have the potential to affect the cardiovascular responses (Fico et al., 2022). Exercise is associated with the central command mechanism and the exercise pressor reflex (Teixeira & Vianna, 2022; Williamson et al., 2006), both of which affect the autonomic responses that are elicited. With apnoea, both the absence of respiratory movements (Angell‐James & De Burgh Daly, 1969; Persson et al., 2023) and the gradual development of hypoxia and hypercapnia (Lin et al., 1983a, 1983b; Leuenberger et al., 2001) are important inputs for the cardiovascular responses. Cold‐water face immersion augments the apnoeic bradycardia during both rest and exercise (Andersson & Evaggelidis, 2009; Andersson & Schagatay, 1998a), via activity of a trigeminal–brainstem–vagal reflex (Khurana et al., 1980), showing that cold thermoreceptors in the face are providing input that affects the cardiovascular diving response. To what extent the partly conflicting cardiovascular responses from exercise, absence of respiratory movements, activation of chemoreflexes and activation of the trigeminal cold‐stimulation reflex interact in a non‐steady‐state (i.e., during exercise and apnoea initiated simultaneously) has not been addressed in any previous study.

The present study concerns cardiovascular, respiratory and splenic responses to different combinations of exercise, rebreathing, apnoea and cold‐water face immersion in healthy individuals. The experimental protocol was designed such that we could address two major aims. First, we wanted to study the effects of apnoea on the physiological responses to exercise by comparing exercise with normal breathing and exercise with rebreathing versus exercise with simultaneously initiated apnoea (dynamic apnoea). The hypothesis was that with increasing duration of apnoea, the typical exercise response would be converted into an apnoea response (diving response). Second, we wanted to study the effects of cold‐water face immersion on the physiological responses to dynamic apnoea. The hypothesis was that, similar to steady‐state conditions (rest or exercise), cold‐water face immersion would contribute to the development of relative bradycardia, a reduced cardiac output and preservation of the pulmonary oxygen store.

## 2 MATERIALS AND METHODS

### 2.1 Ethical approval

All trials were conducted in conformity with the principles of the *Declaration of Helsinki*. The protocol was reviewed and approved by the Swedish Ethical Review Authority (2022‐04298). With the recruitment of participants, they were provided with written information about the study, such as the procedures, potential risks, and handling of data. At the laboratory, after verbal clarification of trial procedures and potential risks involved, the participants provided their oral and written informed consent to participate in this study. Exclusion criteria were age <18 or >55 years, any known acute or chronic disease, use of medications (except for contraceptives), and pregnancy or attempting to become pregnant. Anomalies in blood pressure, lung function or ECG, measured and evaluated at the beginning of each trial, would lead to discontinuation of the trial.

### 2.2 Participants

Twenty healthy volunteers (17 male and 3 female) were recruited for the study (Table 1). None of the participants was a regular user of tobacco products. One was an occasional smoker, and another occasionally used ‘Swedish snus’ (an oral smokeless tobacco product), but not within at least 24 h before the trial. All participants had some previous experience of breath‐hold diving or apnoea, and 16 subjects were active breath‐hold divers or underwater‐rugby players. Their mean (SD, range) self‐reported maximal breath‐holding time was 234 (80, 60–380) s, and for active breath‐hold divers or underwater‐rugby players, their breath‐holding experience was 9 (11, 0–32) years. The participants were instructed to arrive at the laboratory after ≥2 h without any heavy meal or caffeine‐containing beverages, with only light physical activity being performed within 12 h of the trial.

**TABLE 1:** Characteristics of participants.

Characteristic	Group (*n* = 20)	Female (*n* = 3)	Male (*n* = 17)
Age, years	34 (10, 21–52)	33 (7, 27–40)	34 (10, 21–52)
Height, cm	182 (8, 167–197)	175 (6, 168–179)	183 (9, 167–197)
Body mass, kg	82 (12, 60–103)	71 (9, 63–81)	84 (11, 60–103)
FVC, L	5.4 (0.9, 3.7–7.0)	4.5 (0.7, 3.7–5.0)	5.6 (0.8, 4.3–7.0)
FVC, GLI *z*‐score	0.254 (0.922, −1.083 to 2.909)	0.335 (0.363, −0.070 to 0.632)	0.240 (0.995, −1.083 to 2.909)
RV, L	1.9 (0.4, 1.2–2.6)	1.7 (0.1, 1.5–1.8)	1.9 (0.4, 1.2–2.6)
RV, GLI *z*‐score	0.323 (0.566, −0.602 to 1.426)	0.458 (0.171, 0.300–0.640)	0.299 (0.611, −0.602 to 1.426)

*Note*: Values are the mean (SD, range). FVC and RV were measured seated. Abbreviations: F, female; FVC, forced vital capacity; GLI, global lung function initiative (Quanjer et al., 2012); M, male; RV, residual volume.

### 2.3 Protocol

Upon arrival at the laboratory [ambient conditions: air temperature 22.5 (0.7)°C, barometric pressure 754 (6) mmHg, relative humidity 30.3 (6.2)%], participants received verbal instructions on the experimental protocol and equipment. All participants were encouraged to ask questions before signing an informed consent form. A health questionnaire was completed by each participant, followed by measurements of height and weight. Blood pressure was measured in a seated position, and forced spirometry was conducted with the participant standing. Participants then assumed a supine position on a mattress for a 12‐lead ECG recording. Blood pressure, lung function and ECG data were reviewed to identify any anomalies.

Once cleared for further participation, they transitioned to a seated position on a cycle ergometer (Monark Ergomedic 839E, Monark Exercise AB, Vansbro, Sweden), which they maintained for the remainder of the experimental session. For 15 participants, the left thoracoabdominal, dorsolateral area was exposed (by removing their shirt) to allow ultrasound imaging of the spleen. Participants’ forearms rested on a shelf in front of the ergometer at heart level. A water container (for face immersion trials) was placed between the forearms on the shelf, with water temperature maintained at 10.2 (0.2, 10.0–10.6)°C. Again, the forced vital capacity was measured in order to establish the volume of inspired air during the trials. Thereafter, non‐invasive probes for instruments used during the experimental session (see below) were attached to record continuous cardiovascular data, arterial oxygen saturation and regional oxygen saturation of the right deltoid and rectus femoris muscles. Participants were reminded of key protocol steps requiring special attention, such as procedures immediately before and after each trial. They were instructed to remain as relaxed as possible, avoid voluntary hyperventilation prior to trials, and refrain from performing Valsalva or Müller manoeuvres during apnoeas. The experimental protocol began once stabilized cardiovascular data were observed.

**FIGURE 1:**
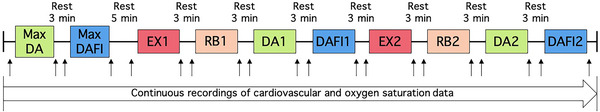
Schematic overview of the experimental protocol. Boxes indicate trial periods, during which 60 W cycle exercise was performed, and horizontal lines in between indicate resting periods between trials. Two maximal‐duration trials (Max DA, maximal dynamic apnoea with the face in the air; and Max DAFI, maximal dynamic apnoea with cold‐water face immersion) were followed by eight submaximal trials (EX, exercise with normal breathing; RB, exercise with rebreathing; DA, dynamic apnoea with the face in the air; and DAFI, dynamic apnoea with cold‐water face immersion). The order of the maximal trials was alternated among participants, and the order of the submaximal trials was randomized among participants. Cardiovascular and oxygen saturation data were recorded throughout the protocol, end‐tidal gases were measured at the start and end of each trial, and spleen dimensions were measured 60 s before and directly after the end of each trial (arrows).

Each participant completed 10 trials (Figure 1). All trials included cycle exercise with a constant workload of 60 W at 40 r.p.m., based on a protocol described previously (Andersson & Evaggelidis, 2009). The first two trials were ‘dynamic apnoeas’ (apnoeic exercise periods). In these trials, the participant began and ended exercise simultaneously with the apnoea, each performed to the maximal duration, once with and once without face immersion in the cold‐water container (order was alternated among participants). No time cues were provided during these maximal‐duration apnoeas. Following these trials, an individual submaximal trial duration was determined collaboratively, based on individual maximal apnoea times; the median difference between individual maximal apnoea times and submaximal trial times was 12 s. The participant then completed eight additional trials, comprising two trials each of eupnoeic exercise (EX), rebreathing exercise (RB), dynamic apnoea without face immersion (DA) and dynamic apnoea with face immersion (DAFI), all at the predetermined submaximal duration. Trial order was randomized for each participant. A 3 min resting period separated each trial, except for a 5 min interval between the last maximal apnoea and first submaximal trial to allow time to discuss the submaximal trial duration.

Trials commenced following a countdown by one of the experimenters. Thirty seconds before each trial, a nose clip was applied, and the participant began breathing through a mouthpiece from an open‐circuit spirometry system to enable end‐tidal gas measurements. During the final 10 s countdown, the participant exhaled to residual volume through the open‐circuit spirometry mouthpiece, then immediately switched to a mouthpiece connected to a prefilled rubber bladder containing a volume of ambient air equivalent to 85% of the seated vital capacity. The completed inhalation of this volume of air marked the start of the trial. For EX trials, the second mouthpiece was removed, allowing free breathing of ambient air. During RB trials, the mouthpiece remained in place, and the participant respired freely into and from the rubber bladder without consciously altering the ventilatory pattern. In the DA and DAFI trials, the rubber bladder mouthpiece was removed, and the participant held the inhaled volume of air in the lungs until the end of apnoea. During DA, the face was held above the water surface, whereas during DAFI the entire face, including the chin and forehead, was immersed into the cold water.

Throughout the submaximal trials, the participant received time cues, and a countdown was given during the final 10 s of the trials. Towards the end of the countdown ending EX trials, the participant inhaled deeply and performed a short‐lasting apnoea while the mouthpiece of the open‐circuit spirometry system was put in place, allowing the participant to end the trial with a maximal expiration on command from the experimenter. Likewise, during the countdown ending RB, the participant inhaled all the gas remaining in the rebreathing bag and performed a short‐lasting apnoea while switching to the mouthpiece of the open‐circuit spirometry system, ending the trial with a maximal expiration on command from the experimenter. Likewise, during the countdown ending DA and DAFI, the mouthpiece of the open‐circuit spirometry system was inserted into the participant's mouth while still holding the breath, ending the apnoea with a maximal expiration on command from the experimenter. The mouthpiece and the nose clip were removed, and the participant rested, remaining in the seated position between trials. After DAFI, the participant's face was dried with a towel, removing any residual cold water.

Beginning 5 min after the last trial, triplicate measurements of the residual volume in the seated position were performed using a nitrogen‐dilution technique (Rahn et al., 1949). In short, after a maximal exhalation to residual volume, the participant rebreathed five times through the open‐circuit spirometry mouthpiece, which at this point was connected to a rubber bladder initially containing 3 L of 100% O_2_. The dilution of inert gas in this closed system, measured in the third exhalation, was used for calculation of the residual volume.

### 2.4 Measurements and data collection

A wall‐mounted height measurer and an electric scale (BF214, Omron Healthcare Europe, Hoofddorp, the Netherlands) were used to measure height and weight, respectively. Pre‐trial blood pressure in the seated, resting position was measured using an automatic sphygmomanometer (Boso‐medicus, Bosch + Sohn, Jungingen, Germany). A hand‐held spirometer (Micro Plus, Micro Medical, Rochester, UK) was used for spirometry measurements in both the standing and seated positions. Glossopharyngeal insufflation, a technique commonly used by competitive freedivers to increase the volume of air in the lungs (Fitz‐Clarke, 2018), was not allowed during spirometry or the rest of the protocol, because this practice is associated with potential adverse effects (Andersson et al., 2009; Chung et al., 2010; Linér & Andersson, 2010). An ECG monitor (Cardiovit AT‐1 G2, Schiller, Doral, FL, USA) was used for recording the 12‐lead ECG prior to further testing.

During the trials, HR, SV, cardiac output (CO), total peripheral resistance (TPR) and arterial blood pressures were recorded continuously using a finger photoplethysmograph (Finapres NOVA, Finapres Medical Systems BV, Enschede, the Netherlands). Methodological considerations regarding the Finapres NOVA monitoring system in similar experimental conditions have been discussed in a previous study (Persson et al., 2023). Briefly, the system records finger arterial pressure using a finger cuff that was placed on the middle phalanx of the right middle finger, with a built‐in photoplethysmograph, calibrated using a blood pressure arm cuff placed over the right brachial artery. The finger pressure is reconstructed into brachial arterial pressure. The Finapres NOVA uses the Modelflow algorithm to calculate cardiovascular variables, such as SV, CO and TPR, from the recorded arterial pressure. In addition to these variables, the skin blood flow (SkBF) was continuously recorded using a laser‐Doppler flowmeter (Advanced Laser Flowmeter 21, Advance Company, Tokyo, Japan). The probe was attached with adhesive tape to the medial side of the distal phalanx of the right thumb. In eight of the participants, the signal quality of the flowmeter was not sufficient for reliable analysis; therefore, the SkBF analysis is based on results from 12 participants.

The arterial haemoglobin oxygen saturation ($S_{aO_{2}}$) was recorded continuously using a finger pulse oximeter (Biox 3700e, Ohmeda, Madison, WI, USA), with the probe placed on the right index finger. As a safety precaution (Linér & Andersson, 2009), one of the experimenters continuously monitored the pulse oximeter during interventions in order that hypoxic interventions (RB, DA and DAFI) could be interrupted if the $S_{aO_{2}}$ were to reach the predetermined termination level of 60%. This level was set based on observations of alveolar gas compositions in apnoeas causing hypoxic loss of motor control in competitive breath‐hold divers (Lindholm & Lundgren, 2006). Regional muscle oxygen saturation was recorded every 4 s using near‐infrared spectroscopy (Nonin SenSmart Model X‐100 Universal Oximetry System, Nonin Medical, Plymouth, MN, USA), with adhesive probes (SenSmart Equanox 8204CA rSO2 sensor, Nonin Medical, Plymouth, MN, USA) attached to the skin above the regions of interest. One sensor was placed above the right deltoid muscle, 5 cm below the acromion, for recordings of the deltoid muscle oxygen saturation ($S_{dO_{2}}$). Another sensor was placed above the belly of the right rectus femoris muscle for recordings of the rectus femoris muscle oxygen saturation ($S_{\mathrm{rf}O_{2}}$).

Expiratory O_2_ and CO_2_ fractions were recorded using an open‐circuit spirometry system (Ergocard Professional, Medisoft, Sorinnes, Belgium). The open‐circuit spirometry system was calibrated using a 3 L syringe (Hans Rudolph, Shawnee, KS, USA) and certified gases (Linde Gas, Solna, Sweden) prior to the start of each experimental session. Temperature, barometric pressure and humidity were measured in the laboratory immediately before each experimental session, and the temperature of both the ambient air and the water container used for face immersions was noted immediately before each trial. From the recorded expired gas fractions and ambient pressure, end‐tidal partial pressures of O_2_ and CO_2_ ($P_{\mathrm{ET}O_{2}}$ and $P_{\mathrm{ETC}O_{2}}$) were calculated.

Ultrasound imaging of the spleen was performed on 15 of the participants, using an ultrasonic device (Sonosite M‐Turbo, FUJIFILM Sonosite Inc., Bothell, WA, USA), with the transducer C60x/5–2 MHz (Transducers, Sonosite Inc.). Ultrasound imaging of the spleen was performed by an experienced sonographer (A.L.‐S.) at 60 s before the initiation of each trial and at the end of all trials. Two images were taken at each time point to determine maximal length (*L*), maximal thickness (*T*) and maximal width (*W*) of the spleen. Splenic volume was subsequently calculated from the collected measurements of *L*, *T* and *W* using the Pilström equation (Lodin‐Sundström, 2015): *L*π(*WT* − *T* ^2^)/3.

The recordings of cardiovascular and respiratory variables began 2 min prior to the first trial and were run continuously until 2 min after the end of the last trial using a data acquisition system (MP100 hardware and AcqKnowledge software, BIOPAC Systems, Goleta, CA, USA), and the data were stored for later analysis.

### 2.5 Data analysis

For each participant, baseline eupnoeic mean values for cardiovascular variables [HR, SV, CO, TPR and mean arterial blood pressure (MAP)], $S_{aO_{2}}$, $S_{dO_{2}}$ and $S_{\mathrm{rf}O_{2}}$ were calculated from the periods 60–30 s prior to the submaximal duration trials. Also, the mean values for cardiovascular variables, $S_{dO_{2}}$ and $S_{\mathrm{rf}O_{2}}$ were calculated from the period 20–10 s before the end of each submaximal trial. This latter period was chosen because we wanted to use a trial period that was as unaffected as possible by the procedures included in the protocol towards the end of the trials (e.g., countdown, respiratory acts, and fitting of the mouthpiece of the open‐circuit spirometry system into the mouth). For $S_{aO_{2}}$, the nadir in the 0–60 s post‐trial period was determined. For all these variables, the trial values were compared with the baseline, eupnoeic values. The relative change from baseline was also calculated for each variable and trial, and results from EX, RB, DA and DAFI were compared between trials. Likewise, mean splenic volume measured from data collected 60 s before the initiation of each trial was used as a baseline and compared with the splenic volume determined at the end of EX, RB, DA and DAFI. Both absolute splenic volume and relative changes in volume during the trials were analysed and compared between trials.

The $P_{\mathrm{ET}O_{2}}$ and $P_{\mathrm{ETC}O_{2}}$ were determined from the last expiration before trials and the first expiration that ended trials. Pulmonary gas exchanges during RB, DA and DAFI were calculated from the differences between volumes of O_2_ and CO_2_ in the lungs at the beginning and at the end of trials. The volume of O_2_ in the lungs at the beginning of trials was calculated by adding the volume of O_2_ in the rubber bladder to the volume of O_2_ in the residual volume. The latter was obtained using the measured end‐tidal fraction of O_2_ in the last, maximal expiration prior to each trial. The same calculations were done for the volumes of CO_2_ and inert gases. For determination of the lung volume at the end of trials, it was assumed that the volume of inert gases in the lungs was constant during trials (Hong et al., 1971; Linér et al., 1993). The O_2_ and CO_2_ volumes in the lungs at the end of trials were subsequently calculated using the end‐trial lung volume and the end‐tidal O_2_ and CO_2_ fractions of the maximal expiration following each trial. For determinations of the breath‐holding time, the recorded traces of expiratory O_2_ and CO_2_ fractions were used (Girardi et al., 2023), and the times required for inhalation from and exhalation to residual volume were included. The differences in volumes of O_2_ and CO_2_ in the lungs at the beginning and at the end of trials, divided by the corresponding measured trial time, gave the pulmonary gas exchanges, representing the alveolo‐capillary O_2_ and CO_2_ transfer (alveolar gas exchange) during these trials.

For each participant, one pre‐trial baseline mean value and mean values from the two trials of each type were calculated. IBM SPSS Statistics, v.29.0.2.0 (IBM Corp., Armonk, NY, USA), was used to perform statistical analysis. Data were checked for normal distribution, using the Shapiro–Wilk test, before further statistical tests were performed. For data following a normal distribution, absolute values for the baseline and trial periods, in addition to the relative changes during trials compared with baseline, were analysed using one‐way repeated‐measures ANOVA with consideration of Mauchly's test of sphericity, followed by Bonferroni‐corrected pairwise comparisons. For data not following a normal distribution, the corresponding values were analysed using Friedman tests with Bonferroni‐corrected Wilcoxon signed rank test. The level used for accepting significance was *P* < 0.05. Corrected *P*‐values > 1.0 are reported as 1.0. Values reported in the text are the mean (SD), unless otherwise stated.

## 3 RESULTS

### 3.1 Trial durations

The initial maximal‐duration apnoeas lasted, on average, 74 (20) s, with a range of 41–128 s. Based on these performances, the submaximal trial duration was set to 66 (17) s, with a range of 40–110 s.

### 3.2 Cardiovascular responses

Pre‐trial, baseline values for cardiovascular variables are presented in Table 2, with baseline values for $S_{aO_{2}}$, $S_{dO_{2}}$, $S_{\mathrm{rf}O_{2}}$ and the volume of the spleen. There were no significant differences between the periods preceding the different types of trials, and therefore one grand mean baseline value for each variable was used for subsequent analysis.

**TABLE 2:** Baseline values for cardiovascular variables, oxygen saturations and splenic volume.

Variable	Baseline mean (SD)
HR, beats min^−1^	75.9 (15.0)
SV, mL	86.7 (24.9)
CO, L min^−1^	6.5 (1.6)
TPR, mmHg min L^−1^	15.6 (10.4)
SkBF, LD units	16.2 (5.3)
MAP, mmHg	108.0 (12.5)
$S_{aO_{2}}$, %	98.0 (1.0)
$S_{dO_{2}}$, %	75.8 (5.6)
$S_{\mathrm{rf}O_{2}}$, %	69.0 (4.4)
Splenic volume, mL	223.2 (89.6)

*Note*: Values are the mean (SD), *n* = 20 (SkBF, *n* = 12; splenic volume, *n* = 15). Cardiovascular and saturation data were recorded 60–30 s, and splenic volume was measured at 60 s, before the onset of the submaximal trials. Abbreviations: CO, cardiac output; HR, heart rate; LD units, arbitrary laser‐Doppler units; MAP, mean arterial blood pressure; $S_{aO_{2}}$, arterial haemoglobin oxygen saturation; SkBF, skin blood flow; S_m_O_2_, deltoid muscle oxygen saturation; $S_{\mathrm{rf}O_{2}}$, rectus femoris muscle oxygen saturation; SV, stroke volume; TPR, total peripheral resistance.

**FIGURE 2:**
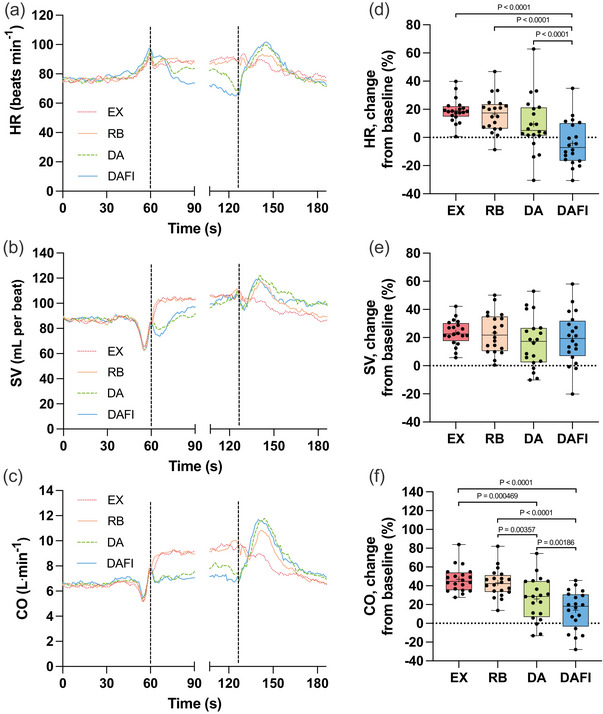
Heart rate (HR; *n* = 20), stroke volume (SV; *n* = 20) and cardiac output (CO; *n* = 20) in association with exercise with normal breathing (EX), exercise with rebreathing (RB), dynamic apnoea with the face in the air (DA) and dynamic apnoea with cold‐water face immersion (DAFI). (a–c) The mean of each variable from before trials (0–60 s), during the first 30 s of trials (61–90 s), during the last 20 s of trials (107–126 s) and during the first 60 s after trials (127–186 s). Vertical lines indicate the start and end of trials. Breaks in the lines reflect the fact that trial durations varied among the participants, and the position of the end of trials in the graphs has been adjusted for each participant to match the average duration of the trials (66 s). Error bars have been omitted for clarity. (d–f) Boxplots for relative changes from baseline during the period 20–10 s prior to ending the trials for each variable. The boxes indicate the first and third quartiles of the data, with the median (horizontal line) and mean (+) values indicated. The whiskers indicate the lowest and the highest data points in the data set. HR: *P* < 0.0001 EX versus DAFI, *P* < 0.0001 RB versus DAFI and *P* < 0.0001 DA versus DAFI; CO: *P* = 0.000469 EX versus DA, *P* < 0.0001 EX versus DAFI, *P* = 0.00357 RB versus DA, *P* < 0.0001 RB versus DAFI and *P* = 0.00186 DA versus DAFI.

During EX and RB, there were increases in HR compared with pre‐trial baseline (Figure 2a). During the period of 20–10 s prior to ending these trials, the HR had increased to 89.8 (16.3) beats min^−1^ (EX, *P* = 0.000886 vs. baseline) and 87.2 (12.7) beats min^−1^ (RB, *P* = 0.00254 vs. baseline), respectively. During the trials involving apnoeas, the initial increase in HR gradually developed into bradycardic responses with the continuation of the trials, such that towards the end of the trials, the absolute HR did not differ significantly from baseline [DA: 81.8 (17.5) beats min^−1^; DAFI: 71.6 (13.1) beats min^−1^; *P* = 0.479 and *P* = 1.0 vs. baseline, respectively]. The analysis of relative changes in HR during the trials revealed that during DAFI, the change from baseline differed from the other three types of trials (Figure 2d), indicating that face immersion augmented the bradycardic response.

We observed rapid increases in SV with the initiation of the EX and RB trials, increases that were maintained throughout the duration of these trials (Figure 2b). Towards the end of these trials, the SV was 106.1 (27.1) mL per beat (EX, *P* < 0.0001 vs. baseline) and 105.5 (27.4) mL per beat (RB, *P* < 0.0001 vs. baseline). As for HR, the pattern of changes in SV during the apnoeic trials differed compared with what was observed when breathing movements were maintained. During both DA and DAFI, the SV did not increase as rapidly as during EX and RB but instead displayed a more gradual increase with the continuation of the trials. During the 20–10 s prior to end of DA and DAFI, the SV was 99.1 (25.3) mL per beat (DA, *P* = 0.0139 vs. baseline) and 101.7 (26.9) mL per beat (DAFI, *P* = 0.00600 vs. baseline), respectively. Considering the relative changes from baseline, there were no differences in the SV changes among the different trials (Figure 2e).

With the increase in both HR and SV during the EX and RB trials, there were rapid and stable increases in CO in these trials (Figure 2c). During the period analysed towards the end of the trials, the CO was 9.5 (2.4) L min^−1^ (*P* < 0.0001 vs. baseline) and 9.2 (2.4) L min^−1^ (*P* < 0.0001 vs. baseline) during EX and RB, respectively. During DA, the CO was not as elevated, 8.0 (1.9) L min^−1^, but still higher than baseline (*P* = 0.000722 vs. baseline). During DAFI, the CO did not increase to a level that differed from baseline, 7.2 (1.7) L min^−1^ (*P* = 0.166 vs. baseline). The relative change in CO was lower during DA compared with EX and RB, and even lower during DAFI (Figure 2f).

**FIGURE 3:**
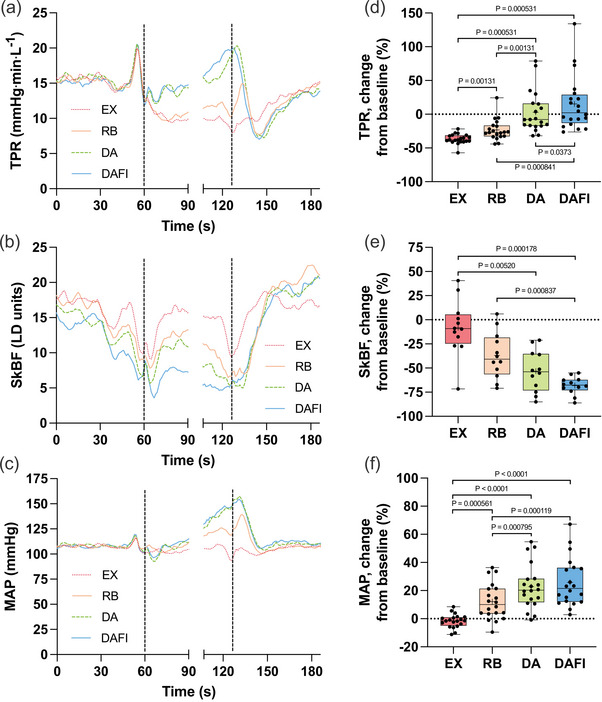
Total peripheral resistance (TPR; *n* = 20), skin blood flow [SkBF, arbitrary laser‐Doppler (LD) units; *n* = 12] and mean arterial blood pressure (MAP; *n* = 20) in association with exercise with normal breathing (EX), exercise with rebreathing (RB), dynamic apnoea with the face in the air (DA) and dynamic apnoea with cold‐water face immersion (DAFI). (a–c) The mean of each variable from before, during and after the trials. (d–f) Shows boxplots for relative changes from baseline for each variable. For details, see the legend to Figure 2. TPR: *P* = 0.00131 EX versus RB, *P* = 0.000531 EX versus DA, *P* = 0.000531 EX versus DAFI, *P* = 0.00131 RB versus DA, *P* = 0.000841 RB versus DAFI and *P* = 0.0373 DA versus DAFI; SkBF: *P* = 0.00520 EX versus DA, *P* = 0.000178 EX versus DAFI and *P* = 0.000837 RB versus DAFI; MAP: *P* = 0.000561 EX versus RB, *P* < 0.0001 EX versus DA, *P* < 0.0001 EX versus DAFI, *P* = 0.000795 RB versus DA and *P* = 0.000119 RB versus DAFI.

During EX, there was a reduction in TPR, 9.8 (6.5) mmHg min L^−1^ (*P* = 0.000886 vs. baseline, Figure 3a). There was a reduction also during RB, 11.7 (6.6) mmHg min L^−1^ (*P* = 0.00780 vs. baseline), which was not as great a reduction as that observed during EX. During DA and DAFI, the reduction in TPR induced by EX was reversed, meaning that towards the end of these trials, the trends of changes were rather that of increases in TPR with time [DA, 15.4 (9.2) mmHg min L^−1^; DAFI, 16.8 (8.9) mmHg min L^−1^; both *P* = 1.0 vs. baseline]. The relative change in TPR differed between DA and EX and RB, and the relative change in TPR during DAFI differed from the changes observed during all the other types of trials (Figure 3d), again indicating that face immersion augmented the response initiated by apnoea.

Alongside the changes in TPR, there were changes in SkBF (Figure 3b). The SkBF was maintained at or close to the baseline during EX, 15.0 (6.3) laser‐Doppler (LD) units (*P* = 1.0 vs. baseline). During RB, the SkBF was lower than baseline towards the end of the trial, 10.6 (5.9) LD units (*P* = 0.00149 vs. baseline). There was a similar change in SkBF during DA, 8.0 (5.6) LD units (*P* < 0.0001 vs. baseline). Throughout the entire DAFI, the SkBF was lower than the baseline, and during the 20–10 s prior to the end, the SkBF was 5.3 (2.8) LD units (*P* < 0.0001 vs. baseline). The relative changes in SkBF were greater during DA and DAFI than during EX, and the reduction in SkBF was greater during DAFI than during RB (Figure 3e).

During EX, the MAP stayed near the pre‐trial, baseline level, 106.3 (13.7) mmHg (*P* = 1.0 vs. baseline; Figure 3c). However, in RB, DA and DAFI, during the period analysed towards the end of the trials, the MAP had increased compared with baseline. During RB, the MAP was 121.0 (19.5) mmHg (*P* = 0.00780 vs. baseline), during DA it was 132.5 (23.6) mmHg (*P* = 0.00103 vs. baseline), and during DAFI it was 135.7 (24.4) mmHg (*P* < 0.0001 vs. baseline). The relative changes in MAP were greater during DA and DAFI than during EX and RB (Figure 3f), but there was no difference between DA and DAFI, i.e., face immersion did not augment the increase in MAP induced by DA.

### 3.3 Arterial and muscle oxygen saturations

**FIGURE 4:**
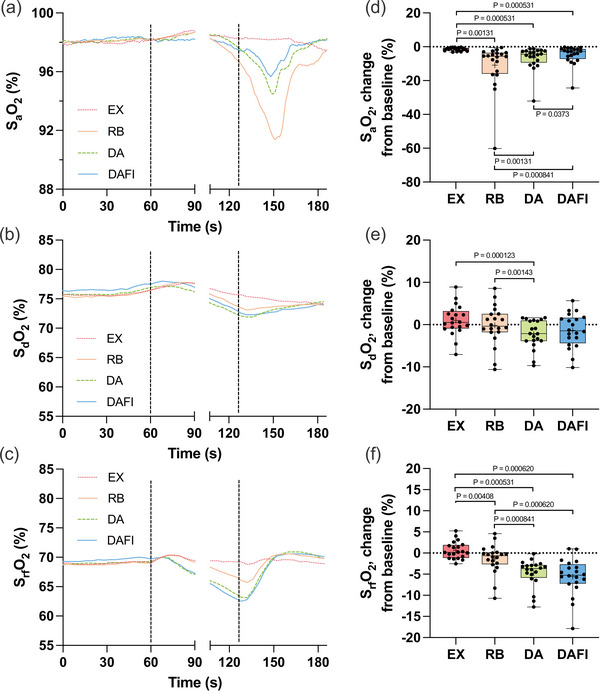
Arterial haemoglobin oxygen saturation ($S_{aO_{2}}$; *n* = 20), deltoid muscle oxygen saturation ($S_{dO_{2}}$; *n* = 20) and rectus femoris muscle oxygen saturation ($S_{\mathrm{rf}O_{2}}$; *n* = 20) in association with exercise with normal breathing (EX), exercise with rebreathing (RB), dynamic apnoea with the face in the air (DA) and dynamic apnoea with cold‐water face immersion (DAFI). (a–c) The mean of each variable from before, during and after the trials. (d–f) Boxplots for relative changes from baseline for each variable. For details, see the legend to Figure 2. $S_{aO_{2}}$: *P* = 0.00131 EX versus RB, *P* = 0.000531 EX versus DA, *P* = 0.000531 EX versus DAFI, *P* = 0.00131 RB versus DA, *P* = 0.000841 RB versus DAFI and *P* = 0.0373 DA versus DAFI; $S_{dO_{2}}$: *P* = 0.000123 EX versus DA and *P* = 0.00143 RB versus DA; $S_{\mathrm{rf}O_{2}}$: *P* = 0.00408 EX versus RB, *P* = 0.000531 EX versus DA, *P* = 0.000620 EX versus DAFI, *P* = 0.000841 RB versus DA and *P* = 0.000620 RB versus DAFI.

Pre‐trial baseline values for $S_{aO_{2}}$, $S_{dO_{2}}$ and $S_{\mathrm{rf}O_{2}}$ are presented in Table 2. The $S_{aO_{2}}$ was maintained at the baseline level during EX (Figure 4a), but on average the minimum $S_{aO_{2}}$ value in the post‐trial period used for analysis was slightly lowered to 96.6 (1.6)% (*P* < 0.0001 vs. baseline). With increasing duration of RB, DA and DAFI, the $S_{aO_{2}}$ was gradually reduced from the pre‐trial baseline level, with the greatest reduction being observed with RB. After RB, the $S_{aO_{2}}$ was reduced to 87.3 (13.2)%, and after DA and DAFI the minimum values were 91.6 (6.8)% and 93.4 (5.5)%, respectively (all *P* < 0.0001 vs. baseline). The relative reduction in $S_{aO_{2}}$ was greater during RB than during DA and DAFI (Figure 4d). There was also a greater reduction during DA than during DAFI.

Expressed in absolute values, the $S_{dO_{2}}$ did not change from baseline during the period 20–10 s before the end of any of the submaximal trials (Figure 4b). During this period, the $S_{dO_{2}}$ was 76.5 (5.7)%, 75.6 (5.5)%, 74.0 (5.1)% and 74.7 (5.4)% during EX, RB, DA and DAFI, respectively (*P* = 1.0, *P* = 1.0, *P* = 0.0949 and *P* = 1.0 vs. baseline, respectively). However, the $S_{dO_{2}}$ was lower during DA than during EX (*P* < 0.001) and RB (*P* = 0.0298). Therefore, when expressed as relative changes (Figure 4e), the change during DA differed from those observed during EX and RB.

During EX and RB, similar to what was observed for the $S_{dO_{2}}$, the $S_{\mathrm{rf}O_{2}}$ did not change compared with baseline (Figure 4c). During the period 20–10 s before ending these trials, the $S_{\mathrm{rf}O_{2}}$ was 69.4 (4.4)% for EX and 67.9 (4.0)% for RB (*P* = 1.0 and *P* = 0.817 vs. baseline, respectively). However, in the same period during both DA and DAFI the $S_{\mathrm{rf}O_{2}}$ was reduced to 65.7 (3.9)% and 65.0 (3.6)%, respectively (*P* < 0.0001 and *P* = 0.000310 vs. baseline, respectively). Expressed as a change from baseline (Figure 4f), RB differed from EX, and both DA and DAFI differed from both EX and RB, without an observed difference between the two apnoeic conditions.

### 3.4 Pulmonary gas exchange

The recordings of ventilation and end‐tidal gases included in the protocol did not allow for analysis of the gas exchange during the resting periods in between trials or during the EX, but analysis was applicable to the RB, DA and DAFI. During RB, when the cardiovascular changes were similar to those observed during EX, the pulmonary oxygen uptake (V˙O2/V˙O2kgkg) was 9.7 (2.3) mL min^−1^ kg^−1^. During DA, the V˙O2/V˙O2kgkg was lower, at 9.1 (2.3) mL min^−1^ kg^−1^ (*P* < 0.0001 vs. RB). The V˙O2/V˙O2kgkg was even lower during DAFI, being 8.8 (2.2) mL min^−1^ kg^−1^ (*P* < 0.0001 and *P* = 0.000569 vs. RB and DA, respectively). As for pulmonary carbon dioxide elimination (V˙CO2/V˙CO2kgkg), during RB it was 3.4 (0.9) mL min^−1^ kg^−1^. This was higher than the V˙CO2/V˙CO2kgkg during DA and DAFI, 3.2 (0.8) and 3.2 (0.8) mL min^−1^ kg^−1^, respectively (*P* = 0.00600 for RB vs. DA; *P* = 0.00104 for RB vs. DAFI; *P* = 1.0 for DA vs. DAFI). With the observed pulmonary gas exchanges, the respiratory exchange ratios (RER) were 0.36 (0.09), 0.37 (0.10) and 0.38 (0.11) for RB, DA and DAFI, respectively. Hence, the highest RER was observed with DAFI and the lowest during RB (*P* = 0.0845 for RB vs. DA; *P* = 0.000578 for RB vs. DAFI; *P* = 0.00809 for DA vs. DAFI).

The $P_{\mathrm{ET}O_{2}}$ and $P_{\mathrm{ETC}O_{2}}$ (Table 3) measured in the final expiration immediately prior to initiating trials were slightly higher and lower, respectively, compared with what is usually considered normal, indicating some form of pre‐trial hyperventilation. In the expiration ending EX, the end‐tidal gases were in the expected normal range. However, for the three other trials, involving an interruption of external gas exchange, the $P_{\mathrm{ET}O_{2}}$ at the end of the trial was lower than the pre‐trial and EX values, and the $P_{\mathrm{ETC}O_{2}}$ at the end of the trial was higher than the pre‐trial and EX‐values. It was also observed that RB resulted in the most pronounced hypoxia and hypercapnia, whereas DAFI resulted in the least hypoxia. For $P_{\mathrm{ETC}O_{2}}$, there was no difference between DA and DAFI.

**TABLE 3:** End‐tidal partial pressures of O_2_ and CO_2_ ($P_{\mathrm{ET}O_{2}}$ and $P_{\mathrm{ETC}O_{2}}$) before and after the submaximal trials.

		END			
Variable	Pre‐trial	EX	RB	DA	DAFI
$P_{\mathrm{ET}O_{2}}$ (mmHg)	116.7 (5.0)	103.1 (7.4)^a^	50.3 (11.9)^[Link]^, ^[Link]^	57.9 (14.0)^[Link]^, ^[Link]^, ^[Link]^	61.4 (13.6)^[Link]^, ^[Link]^, ^[Link]^, ^[Link]^
$P_{\mathrm{ETC}O_{2}}$ (mmHg)	30.3 (3.9)	35.3 (3.1)^a^	51.7 (4.1)^[Link]^, ^[Link]^	49.4 (4.6)^[Link]^, ^[Link]^, ^[Link]^	48.9 (4.5)^[Link]^, ^[Link]^, ^[Link]^

*Note*: Values are the mean (SD), *n* = 20, from the last expiration before trials (Pre‐trial) and the first expiration ending trials (END). Abbreviations: DA, dynamic apnoea with the face in the air; DAFI, dynamic apnoea with cold‐water face immersion; EX, exercise with normal breathing; RB, exercise with rebreathing.

^a^ *P* < 0.0001 vs. Pre‐trial.

^b^ *P* < 0.0001 vs. EX.

^c^ *P* < 0.0001 vs. RB.

^d^ *P* = 0.000753 vs. DA.

^e^ *P* = 0.00267 vs. RB.

### 3.5 Splenic volume

**FIGURE 5:**
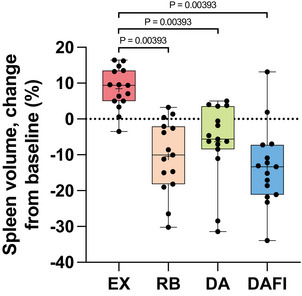
Boxplot for relative changes from baseline in splenic volume (*n* = 15) during exercise with normal breathing (EX), exercise with rebreathing (RB), dynamic apnoea with the face in the air (DA) and dynamic apnoea with cold‐water face immersion (DAFI). For details, see the legend to Figure 2. *P* = 0.00393 EX versus RB, *P* = 0.00393 EX versus DA and *P* = 0.00393 EX versus DAFI.

The pre‐trial baseline volume of the spleen is presented in Table 2. During EX, there was an increase in the volume of the spleen to 239.6 (91.7) mL (*P* < 0.001 vs. baseline). During DA, the change in splenic volume to 206.4 (80.5) mL, compared with baseline was not significant (*P* = 0.323 vs. baseline). However, during both RB and DAFI, we observed significant reductions in splenic volume to 201.8 (90.4) mL and 192.9 (89.4) mL, respectively (RB, *P* = 0.0300 vs. baseline; DAFI, *P* = 0.00114 vs. baseline). Expressed as a relative change in splenic volume from baseline (Figure 5), the response observed during EX differed from those observed during RB, DA and DAFI (all three *P* = 0.00393 vs. EX), without a difference in response among the last three types of trials, either when expressed in absolute values or as relative changes (all *P* = 1.0).

## 4 DISCUSSION

Participants in the study performed trials involving exercise, rebreathing, apnoea and cold‐water face immersion, allowing us to evaluate the cardiovascular, respiratory and splenic responses to these interventions. The central observations reveal that dynamic apnoea with face immersion resulted in the most pronounced diving response, including a bradycardic response and an inhibition of exercise‐induced increase in cardiac output, together with an increase in arterial blood pressure. The cardiovascular adjustments during dynamic apnoea with face immersion were accompanied by a reduction in the delivery of oxygen to the working rectus femoris muscle, which was not observed during rebreathing, and a simultaneously reduced rate of depletion of the central, pulmonary oxygen store. Thus, from a qualitative perspective, most of the physiological responses to dynamic apnoea, observed during the non‐steady‐state when exercise and apnoea are initiated simultaneously, are similar to what is observed with apnoeas performed during rest or steady‐state exercise (Andersson et al., 2004; Bouten et al., 2021; Lindholm et al., 1999; Persson et al., 2023; Tocco et al., 2012).

### 4.1 Characteristics of the included interventions

With the protocol of the present study, the different interventions affected the physiological responses via a multitude of regulatory mechanisms. Initiation of upright dynamic cycle exercise is associated with the central command mechanism, the exercise pressor reflex, local vascular responses, including active hyperaemia, the skeletal muscle pump mechanism and the muscle mechano‐ and metaboreflexes (e.g., Fisher et al., 2015; Hellsten & Nyberg, 2015; Mortensen & Saltin, 2014). In addition to these mechanisms, exercise with rebreathing is associated with activation of chemoreceptor reflexes, owing to the gradual development of hypoxia and hypercapnia (Lin et al., 1983a, 1983b; Persson et al., 2023), but the continued ventilation maintains rhythmic signalling from, for example, pulmonary stretch receptors. This continued pulmonary afferent signalling is absent during dynamic apnoea, allowing any apnoea‐associated brainstem respiratory and cardiovascular control centre interactions that affect cardiovascular reflexes to be activated in this intervention (Foster & Sheel, 2005). Also, when performing apnoea with a lung volume above the functional residual capacity and relaxed respiratory muscles, as in the present study, high intrathoracic pressure could possibly impede venous return and thus reduce the cardiac preload (Andersson & Schagatay, 1998b; Ferrigno et al., 1986). Furthermore, the arterial baroreflex might be reset during dynamic apnoea (Taboni et al., 2021). Finally, in addition to all the mechanisms described above, the dynamic apnoea with cold‐water face immersion possibly affects the physiological responses through afferent signalling from cold thermoreceptors in the region in the face innervated by the ophthalmic division of the trigeminal nerve (Andersson et al., 2002; Khurana et al., 1980).

The interventions included cycle exercise with a workload of 60 W at 40 r.p.m. This means that the workload was not individualized according to the aerobic fitness of each participant, which might be considered as a limitation of the experimental protocol. In the present study, the workload was determined based on earlier tests on a group of trained breath‐hold divers (Andersson & Evaggelidis, 2009). This group of divers were swimming in a pool at the preferred speed they would normally use during dynamic apnoea performances, albeit surface swimming with fins and snorkel breathing, while their heart rates and fin stroke rates were being recorded. Then these divers performed cycle exercise in our laboratory setting (face immersed, snorkel breathing, same water temperature as in the pool) with a workload ‘titrated’ such that their heart rates were similar in both conditions. This resulted in an average cycle ergometer workload of ∼60 W. The fin stroke rate in the pool among this group of divers was also converted to a similar cadence on the ergometer, i.e., 40 r.p.m. Thus, the workload used in the present study mimicked that experienced by trained breath‐hold divers when they perform dynamic apnoea in a pool.

### 4.2 Cardiovascular responses to interventions

As expected, HR, SV and CO increased with the initiation of exercise, and these changes can be ascribed to, for example, the central command mechanism, the skeletal muscle pump mechanism and the muscle mechanoreflexes (Ainab et al., 2025). These cardiac responses were not affected by the gradual development of hypoxia and hypercapnia, with associated activation of chemoreceptor reflexes, because the rebreathing conditions resulted in similar changes from eupnoeic baseline. This illustrates that activation of chemoreceptor reflexes alone, in the absence of apnoea, is not sufficient to initiate the cardiovascular diving response. Similar results have been observed during both rest (Lin et al., 1983a, 1983b; Persson et al., 2023) and steady‐state exercise (Lindholm et al., 1999).

The immediate increases in both SV and CO upon initiation of upright exercise with either eupnoea or rebreathing probably reflect that the skeletal muscle pump promptly increases the venous return, hence the cardiac preload in the upright position (Higginbotham et al., 1986). In the present study, the observed changes in HR, SV and CO were rather stable for the duration of exercise with either eupnoea or rebreathing. Obviously, the addition of apnoea to the protocol affected the cardiac responses. The increase in SV became temporarily impeded during apnoea, but towards the end of the interventions the change in SV did not differ between the different trials. Thus, it seems as if an initial impediment of cardiac preload owing to a high intrathoracic pressure (Ferrigno et al., 1986) was secondarily and gradually counterbalanced by an increase in venous return brought about by the skeletal muscle pump. We can speculate that with time in dynamic apnoea, there will be an accumulation of blood volume in extrathoracic capacitance veins (Mijacika et al., 2017), a venous blood volume that the skeletal muscle pump can act upon, leading to an increase in peripheral venous pressure that gradually restores venous return. This gradual increase in SV is not observed during apnoea in resting, supine individuals (Persson et al., 2023), when there is no effect from the skeletal muscle pump. In the resting, supine situation the SV is maintained at a sub‐eupnoeic level for the duration of apnoea (Persson et al., 2023). It could be argued that a gradually developing bradycardic response during dynamic apnoea (see below) could contribute to the observed increase in SV owing to an increased cardiac filling time (Tocco et al., 2012). However, the bradycardic response is even greater during apnoea in resting subjects without being accompanied by an increase in SV, which lends support to the conclusion that our observation of gradually increased SV during dynamic apnoea should not be attributed to a prolonged cardiac filling time, secondary to a bradycardic response.

The stable tachycardic response observed during exercise with either eupnoea or rebreathing was also affected by apnoea. Towards the end of dynamic apnoeas either with or without cold‐water face immersion, the HR had returned to the resting, eupnoeic level. Thus, the sympathetic, tachycardic response elicited by exercise gradually developed into what can be interpreted as a parasympathetic bradycardic response (Kinoshita et al., 2006; Shamsuzzaman et al., 2014) that became evident with increased duration of dynamic apnoea. This observation falls in line with the observations by Nobihuro et al. (2024), who used apnoea, passive leg cycling and a combination of these to study the extent to which a muscle mechanoreflex would affect the cardiovascular responses to apnoea. In trained breath‐hold divers, although the muscle mechanoreflex was activated by passive leg cycling, apnoea was still associated with a bradycardic response (Nobuhiro et al., 2024). Our observations corroborate these findings and extend them to active dynamic exercise. Thus, it seems that with increased duration of apnoea, the apnoea‐related bradycardia is prioritized over the exercise‐related tachycardia, in line with earlier observations, including simultaneously initiated exercise and apnoea in the protocol (Andersson & Evaggelidis, 2009; Brown et al., 2024; Wein et al., 2007). We also observed that the activation of the trigeminal–brainstem–vagal reflex (Khurana et al., 1980) potentiates the bradycardic response during dynamic apnoea, because the change in HR during dynamic apnoea with cold‐water face immersion differed from all the other interventions. Thus, also in these non‐steady‐state conditions, cold‐water face immersion augments the bradycardic response, similar to what has been established for apnoeas both performed at rest (Kawakami et al., 1967; Schagatay et al., 2007) and during steady‐state exercise (Andersson et al., 2004, 2002).

Dynamic apnoeas also affected the changes in CO that were initiated by exercise. Dynamic apnoea without face immersion resulted in an increase in CO from the eupnoeic level, but the increase was attenuated when compared with the observed changes during exercise with either eupnoea or rebreathing. This can be attributed to the bradycardic response initiated by dynamic apnoea. The increase in CO was attenuated further by cold‐water face immersion, because the CO during the dynamic apnoea with face immersion did not differ from resting eupnoea, and the relative change in CO in this intervention was lower than in all other three interventions. Overall, the cardiac responses to a non‐steady‐state dynamic apnoea are to some extent qualitatively similar to the responses observed in resting apnoeas and in apnoeas performed during steady‐state exercise, but differ in magnitude (Andersson et al., 2025, 2004; Bouten et al., 2021; Costalat et al., 2013; Sivieri et al., 2015; Tocco et al., 2012). The lower CO during dynamic apnoeas will entail both a reduced perfusion of the pulmonary circulation, affecting pulmonary gas exchange and development of arterial desaturation, and a reduced peripheral oxygen delivery, affecting regional oxygen saturations (see section 4.3).

With the observed cardiac changes, we also recorded vascular responses. The TPR was reduced during both exercise and rebreathing, probably reflecting how active hyperaemia and other local regulatory mechanisms contribute to increased skeletal muscle blood flow with the initiation of dynamic exercise (Mortensen & Saltin, 2014). The reduction in TPR was attenuated by rebreathing. This could be interpreted as an effect of chemoreceptor activation, leading to an increase in sympathetic nerve activity causing a relative systemic vasoconstriction (Leuenberger et al., 2001). The SkBF was unaffected by exercise but reduced during rebreathing, probably reflecting the systemic vasoconstriction resulting from rebreathing. Like SkBF, the MAP was unaffected by exercise. Thus, it seems as if the increase in CO is balanced by the decrease in TPR resulting from the low‐intensity exercise of the present protocol. The changes in TPR, SkBF and MAP were all affected by the introduction of apnoea to the protocol. The general vasodilatory response initiated by exercise was reversed into what is interpreted as a systemic vasoconstriction during dynamic apnoeas. With increased duration of dynamic apnoea, the TPR gradually increased, the SkBF was reduced and the MAP increased. The change in TPR from baseline was the greatest during the dynamic apnoea with face immersion, without a difference in MAP between dynamic apnoea with and without face immersion. This can be explained by the lower CO during dynamic apnoea with face immersion, counteracting the higher TPR. Taken together, the recorded vascular responses indicate that dynamic apnoea is associated with systemic vasoconstriction, most probably following increased sympathetic nerve activity (Fagius & Sundlöf, 1986). In comparison to rest (Andersson et al., 2025), the increase in TPR during apnoea appeared to be delayed in onset when apnoea was initiated simultaneously with dynamic exercise. This could reflect that the vasoconstriction response to apnoea is modified by different regulatory mechanisms related to exercise, such as locally acting active hyperaemia and muscle mechano‐ and metaboreflexes. Nevertheless, similar to what was mentioned above with regard to the reduced CO, the systemic vasoconstriction elicited during dynamic apnoea will probably be associated with a reduced peripheral oxygen delivery, secondarily affecting regional oxygen saturations (see below).

### 4.3 Central oxygen stores and peripheral oxygen delivery during interventions

We observed changes in the handling of central and peripheral oxygen stores that we attribute to the cardiovascular responses that were initiated by the interventions. It was not possible to analyse the pulmonary oxygen uptake during the exercise trial. However, given the similarities in cardiovascular responses during exercise and rebreathing, especially given that we observed the same CO during these trials, we assume that the pulmonary oxygen uptake was most likely to be very similar during the exercise and rebreathing interventions. Changes in the pulmonary gas exchange during rebreathing and apnoea are dependent to a great extent on changes in CO (Linér & Linnarsson, 1994; Persson et al., 2023). Thus, the highest pulmonary oxygen uptake in the present study was observed during rebreathing. Both dynamic apnoea without and dynamic apnoea with cold‐water face immersion resulted in a reduction in pulmonary oxygen uptake, with the latter conditions resulting in the lowest pulmonary oxygen uptake. This is reflected by the change in CO observed in each intervention, respectively. Hence, dynamic apnoea with cold‐water face immersion resulted in the lowest CO and lowest pulmonary oxygen uptake. This observation, obtained from a non‐steady‐state protocol, is in line with what has been reported in studies using various forms of steady‐state interventions in their protocols (Andersson et al., 2025, 2004; Lindholm & Linnarsson, 2002; Linér & Linnarsson, 1994).

Given that the cardiovascular responses elicited by dynamic apnoea resulted in a reduced rate of depletion of the pulmonary oxygen store, it is not surprising that the $S_{aO_{2}}$ was reduced to a lesser extent the more the increase in CO was attenuated by the apnoea‐induced cardiovascular responses (Andersson & Schagatay, 1998a; Persson et al., 2023); i.e., the $S_{aO_{2}}$ was the highest after dynamic apnoea with cold‐water face immersion, the intervention during which the CO was not increased by the exercise stimulus, and the HR displayed a bradycardic trend throughout the intervention. The $S_{aO_{2}}$ was the most reduced after rebreathing exercise, when the cardiovascular responses were similar to what was observed during eupnoeic exercise. Thus, it seems that during rebreathing the lack of apnoea‐induced cardiovascular responses at the fastest pace will lead to a level of hypoxia that potentially threatens the function of hypoxia‐sensitive organs, such as the brain and the heart.

Although the central oxygen store was preserved by the apnoea‐induced cardiovascular responses, the opposite happened to the peripheral oxygen stores, as reflected by the changes in regional muscle oxygen saturations that we recorded. The regional muscle oxygen saturation predominantly reflects the venous oxygen store at the site of measurement (Barstow, 2019; Steppan & Hogue, 2014) and provides information on the balance between regional oxygen supply and demand. The changes in $S_{\mathrm{rf}O_{2}}$ are of particular interest. In the present study, the $S_{\mathrm{rf}O_{2}}$ will represent, to a large extent, the saturation of the venous blood in a muscle being activated during the interventions included in the protocol. It is interesting to note that during dynamic apnoea both without and with cold‐water face immersion, when the CO was not increased and the peripheral blood flow most probably was the most reduced secondarily to the increase in TPR, the $S_{\mathrm{rf}O_{2}}$ was quickly and markedly reduced. At the same time, $S_{aO_{2}}$ was maintained to a higher degree during the dynamic apnoeas. This corresponds to a widening of the arterial‐to‐venous difference in oxygen content at the site of measurement during the dynamic apnoeas. This relative depletion of the peripheral oxygen stores during dynamic apnoeas contributes to the preservation of the pulmonary oxygen store because of a simultaneous prolongation of the turnover time of the peripheral venous blood (Andersson et al., 2004; Linér & Linnarsson, 1994). Our observations are in line with earlier studies (Elia et al., 2025; Palada, Obad, et al., 2007; Persson et al., 2023; Valic et al., 2006) concluding that the peripheral vasoconstriction during apnoea will redistribute arterial blood flow, hence oxygen delivery, towards vital, hypoxia‐sensitive organs, such as the brain, while limiting blood flow to muscles and other hypoxia‐tolerant tissues. With this line of reasoning, the apnoea‐induced cardiovascular responses offer an oxygen‐conserving effect, preserving the central, pulmonary oxygen store at the expense of peripheral, venous oxygen stores. Thus, we can expand conclusions about the oxygen‐conserving effects from the human diving response that have been established for steady‐state conditions (Andersson et al., 2008, 2004) to be valid also in non‐steady‐state conditions.

### 4.4 Effects of interventions on splenic volume

During the short‐lasting, low‐intensity eupnoeic exercise included in the protocol of the present study, we observed an increase in splenic volume. We postulate that this increase in splenic volume is secondary to the increase in CO with eupnoeic exercise, causing an increase in splenic artery blood flow, leading to an accumulation of blood volume in the spleen. It is possible that the splenic volume would have been reduced, reflecting a sympathetically mediated splenic contraction, if the exercise period had been prolonged or performed at a higher percentage of maximal oxygen uptake (Lindblom et al., 2024; Stewart et al., 2003).

When expressed as a relative change from baseline, the rebreathing intervention and both types of dynamic apnoea interventions differed in response when compared with the eupnoeic exercise. In fact, both rebreathing and dynamic apnoea with face immersion resulted in a reduction in splenic volume from the volume measured at baseline. The finding that the splenic volume was reduced in the latter condition is in accordance with the observations by Elia et al. (2021). They studied a series of repeated dynamic apnoeas in immersed breath‐hold divers, whereas we studied dynamic apnoeas in non‐immersed subjects. Nevertheless, whole‐body immersion does not seem to be a necessary stimulus for eliciting the apnoea‐induced contraction of the spleen that is responsible for the reduction in splenic volume (Baković et al., 2003; Schagatay et al., 2001), because we observe a reduction in splenic volume in our non‐immersed subjects. With the splenic contraction, there will be a release of erythrocytes that are sequestered in the spleen, which offers protection in different types of hypoxic conditions, including apnoea and high‐altitude exposure (Lodin‐Sundström & Schagatay, 2010; Lodin‐Sundström et al., 2021; Schagatay et al., 2001).

We have previously shown that in resting subjects, rebreathing is not as potent as apnoea in eliciting splenic contraction and the associated increase in haemoglobin concentration in the circulating blood (Persson et al., 2023). It was concluded that the cessation of respiratory movements during apnoea is a major trigger for splenic contraction. In the present study, we observed no difference in the post‐intervention splenic volume between rebreathing and dynamic apnoeas, which was a somewhat surprising observation. However, it should be noted that the rebreathing exercise in the present study was associated with a rapid progression of arterial desaturation leading to a highly pronounced hypoxia at the end of the intervention. In comparison to rebreathing performed at rest (Persson et al., 2023), both the time course and the final level of arterial desaturation in the rebreathing exercise were more severe. Also, when compared with the dynamic apnoeas of the present study, the arterial hypoxaemia occurred earlier and to a greater extent in the rebreathing exercise. Hypoxia, in addition to apnoea itself, is known to be a stimulus for splenic contraction in different conditions (Lodin‐Sundström & Schagatay, 2010; Lodin‐Sundström et al., 2021; Richardson et al., 2009). Furthermore, it is possible that hypercapnia, which developed to a greater extent during the rebreathing exercise than during the dynamic apnoeas, could contribute to stimulate splenic contraction (Elia et al., 2022; Richardson et al., 2012), although the observations in favour of the importance of hypercapnia in this context are not as conclusive as those favouring the importance of hypoxia. Thus, the augmented hypoxia and, to some extent, hypercapnia, in the rebreathing exercise in the present study might have offset the importance of the absence of respiratory movements for the initiation of splenic contraction, resulting in similar splenic volumes after rebreathing exercise and dynamic apnoeas.

## 5 CONCLUSION

In relationship to the aims of the study, the following can be concluded based on the observations. First, with increasing duration of dynamic apnoea, the typical cardiovascular responses to eupnoeic exercise were converted into apnoea responses, indicating that the diving response will have priority over the exercise responses as the apnoea progresses. Second, similar to steady‐state conditions, cold‐water face immersion contributed to the development of relative bradycardia, a reduced cardiac output and a preservation of the pulmonary oxygen store at the expense of peripheral, venous oxygen stores. Thus, also in the non‐steady‐state conditions of the apnoea interventions of the present study, the diving response offers a temporary oxygen‐conserving effect. Finally, we can confirm that dynamic apnoea will be associated with a reduction in splenic volume, but that a reduction in splenic volume was also observed during rebreathing exercise. The physiological responses to non‐steady‐state dynamic apnoea are similar to those elicited by apnoeas at rest or during steady‐state exercise.

## AUTHOR CONTRIBUTIONS

Johan P. A. Andersson contributed to the conception of the study and was, together with Bodil Sjögreen, responsible for project administration and providing resources. All authors contributed to the design of the experimental protocol, data collection, analysis and interpretation towards important intellectual content. Johan P. A. Andersson, Theodore Dotevall and Maja Persson drafted the manuscript. All authors were involved in the revision of the draft, approved the final version of the manuscript manuscript and agree to be accountable for all aspects of the work in ensuring that questions related to the accuracy or integrity of any part of the work are appropriately investigated and resolved. All persons designated as authors qualify for authorship, and all those who qualify for authorship are listed.

## CONFLICT OF INTEREST

None declared.

## FUNDING INFORMATION

This research did not receive any specific grant from funding agencies in the public, commercial, or not‐for‐profit sectors. Funds for open access publication fees were obtained from the Lund University Library and the Faculty of Medicine, Lund University.

## Data Availability

Restrictions in accordance with the approval from the Swedish Ethical Review Authority apply to the availability of the data supporting the conclusions of this study. The data are not publicly available but may be obtained from the authors upon reasonable request and compliance with the ethical approval.
